# Valedictory Address to the Graduating Class in Atlanta Medical College

**Published:** 1879-03

**Authors:** J. S. Boynton

**Affiliations:** Griffin, Ga.


### VALEDICTORY ADDRESS TO THE GRADUATING CLASS IN ATLANTA MEDICAL COLLEGE, MARCH 4, 1879.

By COL. J. S. BOYNTON, Griffin, Ga.

Young Gentlemen:

In behalf of the Faculty, I am authorized to commend your diligence and congratulate you on your progress and success in the collegiate course; to greet you with a cordial welcome to the threshold of the medical fraternity.

Your diplomas are passports and evidences of your respectable entrance among those who have consecrated themselves to that profession which should be esteemed the most honorable of secular callings. Honorable because it is an enquiry into the sublime structure of the human fabric; its various endowments, composition and uses; its diseases, derangements and decays. Watson has wisely remarked, "you cannot have looked into the mechanism of that intricate but perfect work, you cannot have contemplated its fullness of exquisite contrivance, its endless examples of means adjusted to ends, its prospective expedients against future needs, its compensations for inevitable disadvantages, its direct provisions for happiness and enjoyment, without receiving the profoundest conviction of the being and the attributes of its maker. It is upon human anatomy, that Paley in his unrivalled argument for Natural Theology, "takes his stand," and sixteen centuries before him, Galen had felt that, in writing his anatomical treatise, he was composing a hymn to the Deity, that a declaration so pure of the wisdom, power and goodness of God, was an act of piety and praise. Above if not beyond these investigations, is the high object to be accomplished, by wisely assisting nature in throwing off diseases, and, as far as practical, to prevent decay, by the use of such remedies as your scientific learning suggests, are requisite to the purpose of restoring health and relieving pain. To accomplish these noble purposes, it becomes essential for you to acquire a thorough knowledge of chemistry, and be able to grope amid the innumeral minerals, herbs, plants and flowers, which are endowed by divine power with medicinal qualities, tending to cure maladies, relieve suffering, and, in a word, restore health, and be able to administer the most efficacious of these at once. The field of labor is extensive, intricate, inspiring awe in its magnitude. When we pause and contemplate the crushing, or hope inspiring consequences, of your action, in an immergency, on the individual, his family and the community in which you labor, it will provoke a conception of the duties, anxieties and perplexities so intense in character, as to suggest, the thought of an avalanch, moving down upon a community, with no mind to devise, no hand to provide the means of avoiding the approaching danger, but your own. To-night you are brought face to face with these duties, ultimately perhaps with these consequences. Allow me to suggest, that it will require active, personal effort and a heroic individuality, to become victorious in the contest. You may have imagined, that you have reached the goal to which you aspired, when you conceived the idea of being a physician; that you have entered the Promised Land, filled with golden, tinted prospects, ample fountains of wealth and pleasure, from which to quaff every ambitious hope. Indulge in no such delusions. The progress you have made, is but a flower plucked on the highway. Neglect to nurture and care for it by all the appliances, and it withers, dies within the hour, and you will have only worthless leaves, or at best a souvenir of what might have been. Once upon a time, a man by the name of Moses had a vexatious contest with old Pharaoh, before he could induce him to allow his people to quit Egypt and cross over the Red Sea, as you have professionally done to-night; and, for the sake of my learned friends, the professors, I am gratified it is not a literal occurance. When Moses and his people found themselves safely across and free from bondage, they sang praises and gave thanks; but while yet rejoicing, they immerged into a wilderness, and he found himself surrounded by many difficulties and grave responsibilities; still he was firm and endeavored submisively to perform every duty, for forty years, and yet, he only had a view from afar, of the Promised Land. Since we have Moses and you all in the wilderness, it occurs to me, that I can get you out in no safer way, than by giving a little original advice. It is original on the line only of a sentiment which a friend, insisted on giving over a social glass. Says he, before drinking, allow me to offer an original toast: Unique, appropriate, elegant in expression, never conceived by mind, or uttered by lips on any similar occasion. "Old fellow, here's luck." Now I offer as a counterpart, in conception and elegance. Old horse, get out of the wilderness! Get out of the wilderness!

By this seeming ridicule I would not have you understand that I place a low estimate on what has been taught and learned here; far from it. But to impress the idea that this college, although justly renowned for thorough instruction, cannot make any one of you a physician in the practical sense. Colleges do not make men, but learned men, when organized, make colleges. They give you the primary or theoretic instruction, and start you out in the great world with the title of M.D., and by your actual conduct and judicious application of what you have been taught here and afterwards acquire, you may or may not make that title honorable.

These learned and skilled professors have furnished you with what may be termed a topographical map of the ground over which you must travel professionally, on which they have marked out many of the more important highways and obstacles; but the devious ways—the bypaths, little streams, the minutia—must be learned by practical observation, by mixing brains and learning, business and enterprise, and driving all with personal effort. You can thus make the chance for success, and having made it, you can then dart at and gobble up the chance, like a trout on a minnow. An empty exchequer is an oppressive master, requiring thought and diligence. In old age, however, it furnishes no comforts.

We travel, manufacture, plow and transport by steam, correspond by electricity, read our devotions by proxy, accumulate fortunes for loved ones by dying, write by short hand, physic by patent, and learn science by hearing lectures; but as yet we have not invented any process by which one can think for another. Precedents and the experience and observation of others are of vital import, and should be thoroughly learned, carefully digested, and garnered in the store-house of memory, ready, like a bank account, to be drawn on in any emergency. But if any of you are so ignorant or indolent as to contemplate the possibility of relying on what others have thought, seen or written, then burn up your diploma, leave off the M.D., and hire yourself out to break rock before you leave the city.

Be assured only those certainly succeed who are prolific in devices and fertile in expedients—that look on obstacles as things to be vanquished, or regard them as springboards by which to vault across the gulf of failure to the sure, solid ground of success. Our strength is measured by our plastic power. From the same materials one man builds palaces, another one hovels; one warehouses, another villas. Bricks and mortar are mortar and bricks until the builder makes them something else. Then, instead of saying man is the creature of circumstances, it would be nearer the mark to say that man is the architect of circumstances. Nor is it well to be regarded as the promising man; for he who draws largely on expectations will have his drafts returned dishonored. The coming man is a failure; the fellow who wins is already on hand.

Yours is no common-place calling. Your services will be required only on occasions of delicate, doubtful or hazardous character. Often your thoughts and acts, concentrated into a few moments, will determine the health, happiness or life of the patient. At another time your own life may be in as much jeopardy as that of the person visited. Again, you may be called to walk amid the solitude of pestilence and wide-spread lamentations of woe and anguish too terrible for description, too heartrending for contemplation. It would shudder the frame, derange the nerves, overflow the sympathies, and in its presence one would stand transfixed—appalled. Yet, amid such scenes, with such surroundings, you may be called to think, act and alleviate. Stop where you are in preparation; do you believe yourselves equal to such an emergency?

It is not in my power to prescribe any fixed rules of conduct and labor that will qualify you for these duties. The formulas for getting on in the world, which are laid down in maxims, lectures and treatises on that subject, might be enunciated; but men do not conquer by routine no more than a man can learn politeness by practicing the rules prescribed by Lord Chesterfield. In that way one might become formal, but to be polite there must be sympathy, enthusiasm, gushing, in order to have winning ways. Neither can an officer become a great commander by studying the theory and history of war, nor can a lawyer become a great jurist by learning precedents. No more can you rise to eminence as a physician by acquiring what is laid down in the books. Theory and knowledge are indispensable. No business, calling or profession can be well or honorably followed or successfully pursued without them. But there must be an individuality marked, distinct, self-reliant, and, in your profession, heroism, superadded, to drive the man on to success. The engine may be perfect in all its parts, the boiler full of water, the furnace of fuel, still there must be fire to generate steam to start the machine to moving. So thought, practical thought, is the generic power which moves the man—gives him individuality; and this marks him as different from the common herd.

In looking over the world, the relative habits, uses, instincts, designs and purposes of each separate creation are apparent; and we observe how all are beautifully blended into the grand harmony of nature. The landscape presents its dense foliage, plants, grasses, cereals, flowers, rivulets. The hills and valleys present striking peculiarities, while the distant mountain, lifts its lofty peak toward the skies, giving grandeur to the panorama, each contributing manifold beauties to adorn the scene. From these observations we deduce the idea of a striking parallel, between nature and the attributes of humanity. The variety of form and feature, and contrast in person, indicate the natural undulations of hill and vale; but like the mountain, the distinguishing feature of human character, is individuality. It magnifies the power of self and indicates a firm reliance on personal effort; conceives results and suggest ways and means to accomplish; confronts dangers and obstacles, and inspires courage to overcome and conquer. Its attributes are decision of character, fixedness of purpose and enthusiasm, and it is this characteristic which makes the man of progress. If one is content to be a man, simply in resemblance of others, or to move on in the groove made by his ancestry or preceptors, then he is a mere follower. I would stir your ambition to nobler ends. To move magestically along ways of your own construction or choosing—to become thinkers and leaders.

Maury and others have so distinctly designated the highways on the ocean, that one with ordinary intelligence might direct the course of a vessel from one harbor to another, yet it would be reckless indeed, for one with no more knowledge than could be acquired from Maury's great book to undertake to navigate the high seas. Minute information of all the details is requisite for such responsible duties. So it is in your profession, indeed in all a thorough knowledge of details is pre-eminently essential; must depend on every stroke of your pen in writing a prescription; in every word you utter; in suggesting the effects and consequences; how the patient is to be nursed, controlled and fed. Even the intonations of voice and facial expressions are trifles worthy of consideration. Great truths and leading principles must be learned and understood; but without an intricate observance of details you will constantly find yourself embarrassed by difficulties, if not mortified by innumerable failures.

You have gone through the school; to-night you separate from professors and fellows; now indeed you commence the battle of life; if you would gain victory, drill and discipline your forces; learn the various movements from the square drill to the evolutions of the line; enlist new recruits; place the veterans in reserve; organize and have all advantageously posted, so that at any given moment, you may bring them marshalled in good order, promptly into the contest. Bear in mind that you cannot rely on foraging for supplies, like cavalry on a raid. If you do, while foraging or seeking information about how to treat the disease, the patient dies and victory's lost.

You cannot hope for immediate success; indeed you are not prepared for it; wait and labor for the better time, for it will come, if you are diligent and watchful; do not become impatient and halt at the base of Sinai, and begin to gather gold to make an idol of. Leave that ignoble duty for quacks and nostrum peddlers; live up to all the ethics of your profession. Take that which is your due and the accumulations, if husbanded in a business way, will be commensurate with your merit.

Would you have me recite what others have done and accomplished, in order to incite you to diligent action? Rather let me conjure you to contemplate the dignity of your chosen profession, the magnitude of responsibility assumed, the exalted, sacred mission you have entered on. If these fail to move you right onward, then example and precept enforced with matchless eloquence would fail. If one rose from the dead and exhorted you, that would fail. If all the fires of Vesuvius were poured on such a terrapin-like shell, that would fail to produce even a *single crawl.* Would you have me detail how other men have risen? One and all of the successful of the past and present, and those who will rise in the future have, and must, observe the same rule. It is short, inflexible, inexorable—labor and thought—thought and labor, WITH A SOUL IN THE WORK.

In taking leave, young gentlemen, permit me to thank you and this patient audience for attention to the rambling thoughts which I have loosely thrown together in the midst of severe professional engagements. To a great extent the health, life, liberty, property and secrets of the community in which you locate will be entrusted to your keeping. Your mission specially is to heal the sick, relieve pain, make the lame to walk, the blind to see; but there is a higher duty—if you come well up to your duty—which you may perform. While standing by the bedside of affliction feeling the pulsations of the heart with one hand, you may, with the other, point out the way to a higher destiny—the immortality of the soul and the more perfect life beyond. The Saviour was called the Good Physician, and his mission was to go about doing good. You can best fulfil your duty and destiny by imitating his noble example.

